# Hypertension in patients on dialysis: diagnosis, mechanisms, and management

**DOI:** 10.1590/2175-8239-JBN-2018-0155

**Published:** 2018-11-08

**Authors:** Sérgio Gardano Elias Bucharles, Krissia K.S. Wallbach, Thyago Proença de Moraes, Roberto Pecoits-Filho

**Affiliations:** 1 Universidade Federal do Paraná Hospital de Clínicas CuritibaPR Brasil Universidade Federal do Paraná, Hospital de Clínicas, Curitiba, PR, Brasil.; 2 Fundação Pró Renal CuritibaPR Brasil Fundação Pró Renal, Curitiba, PR, Brasil.; 3 Pontifícia Universidade Católica do Paraná Faculdade de Medicina CuritibaPR Brasil Pontifícia Universidade Católica do Paraná, Faculdade de Medicina, Curitiba, PR, Brasil.

**Keywords:** Hypertension, Renal Dialysis, Peritoneal Dialysis

## Abstract

Hypertension (blood pressure > 140/90 mm Hg) is very common in patients undergoing regular dialysis, with a prevalence of 70-80%, and only the minority has adequate blood pressure (BP) control. In contrast to the unclear association of predialytic BP recordings with cardiovascular mortality, prospective studies showed that interdialytic BP, recorded as home BP or by ambulatory blood pressure monitoring in hemodialysis patients, associates more closely with mortality and cardiovascular events. Although BP is measured frequently in the dialysis treatment environment, aspects related to the measurement technique traditionally employed may be unsatisfactory. Several other tools are now available and being used in clinical trials and in clinical practice to evaluate and treat elevated BP in chronic kidney disease (CKD) patients. While we wait for the ongoing review of the CKD Blood Pressure KIDGO guidelines, there is no guideline for the dialysis population addressing this important issue. Thus, the objective of this review is to provide a critical analysis of the information available on the epidemiology, pathogenic mechanisms, and the main pillars involved in the management of blood pressure in stage 5-D CKD, based on current knowledge.

## Introduction

Understanding the mechanisms, evaluating, and defining the best management of blood pressure (BP) in patients receiving renal replacement therapies through hemodialysis (HD) or peritoneal dialysis (PD), is a significant challenge for healthcare professionals. Although BP is measured frequently in the dialysis treatment environment, aspects related to the measurement technique employed may be unsatisfactory. Several other tools are now available and being used in clinical trials and clinical practice to evaluate and treat elevated BP in chronic kidney disease (CKD) patients.^1^^,^^2^ Different levels of BP may be observed in the same patient under distinct situations, which include evaluations before, during, or after the dialysis session, and at home using ambulatory BP measurements (ABPM), being frequently and substantially lower than during dialysis measurements.^3^

In patients with end-stage renal disease (ESRD) receiving dialysis, elevated blood pressure is common and poorly controlled in general.^4^ Although volume overload and sodium retention appear to be the main pathogenic mechanism of hypertension in this population, other factors such as increased arterial stiffness, activation of renin-angiotensin-aldosterone system, sleep apnea, activation of sympathetic nervous system, and use of recombinant erythropoietin may be also involved.^5^

The association between hypertension and cardiovascular disease risk has been well documented in the general population but in dialysis patients the associated risk is poorly understood, and still present paradoxical and unexpected reports.^6^ The presence of stage 5-D CKD is associated with a several-fold increased risk of cardiovascular mortality, compared to age- and sex-matched controls without CKD.^7^ Epidemiological studies have shown that systolic blood pressure (SBP), diastolic blood pressure (DBP) along with traditional risk factors for cardiovascular disease are associated with end-organ damage, including vascular stiffness and poor outcomes in dialysis patients. Indeed, increased and decreased SBP are both associated to cardiovascular disease (CVD) events and decreased SBP following previous hypertension (HTN) is also associated with adverse outcomes.^8^ While we wait for the ongoing review of the CKD Blood Pressure KIDGO guidelines, so far there is no guideline for the dialysis population addressing this important issue. Thus, the objective of this review is to provide a critical analysis of the information available on the epidemiology, pathogenic mechanisms, and the main pillars involved in the management of blood pressure in stage 5-D CKD, based on current knowledge.

### Epidemiology of hypertension in stage 5 CKD dialysis patients

Hypertension (blood pressure > 140/90 mm Hg) is common in patients undergoing regular dialysis, with a prevalence of 70-80% among regular hemodialysis patients^9^ and only the minority has adequate blood pressure control. The scenario for peritoneal dialysis (PD) patients is not different, and the variability reported for the prevalence of hypertension is even higher ranging from approximately 30 to more than 90%.^10^ This variability is mostly related to differences in the definitions used to diagnose hypertension and the tools applied in various studies.^5^ Epidemiological studies in hemodialysis patients in USA, using different ways to define hypertension, revealed that 72 to 88% of all patients studied had elevated BP.^4^^,^^11^^,^^12^ However, in those studies, a high proportion of patients with elevated blood pressure was taking antihypertensive agents and the number of patients with controlled BP was low, between 30-50%.^4^^,^^11^

In contrast to the unclear association between predialytic BP recordings and cardiovascular mortality, prospective studies showed that interdialytic BP, recorded as home BP or by ambulatory blood pressure monitoring in hemodialysis patients, has a clearer association with mortality and cardiovascular events.^13^^), (^14 In a cross sectional study conducted in Italy with patients on peritoneal dialysis using the WHO/ISH definition, the prevalence of elevated BP was 88%. 15 In other studies, the average 24-hour BP was not different between patients on automated peritoneal dialysis and continuous ambulatory peritoneal dialysis^12^^,^^16^ and there were positive correlations of left ventricular mass index with BP measurements and BP load.^16^ Elevated blood pressure diagnosed outside the dialysis unit with home or ambulatory BP monitoring is closely related to mortality.^3^^,^^13^ Additionally, dialysis patients often do not have the normal decrease in BP at nighttime,^17^ increasing their risk for the development of left ventricular hypertrophy and cardiovascular mortality.^18^ Indeed, Foley et al.^19^ observed that each 10 mmHg rise in mean BP was independently associated with a progressive increased prevalence of concentric left ventricular hypertrophy, development of "de novo" cardiac failure, and "de novo" ischemic heart disease. Indeed, the degree of cerebral atrophy and predialytic BP as well as cerebral atrophy and duration of hypertension exhibit very high correlation.^20^ These data suggest that long-term hypertension is frequently, not well controlled, and a significant risk factor for cardiovascular events in CKD hemodialysis patients.

### Diagnosis of hypertension in dialysis patients

The diagnosis of hypertension in the general population is based on different available guidelines, such as the American, Brazilian, and European guidelines, increasing the complexity and controversy of the problem.^21^^-^23 The National Kidney Foundation - Kidney Diseases Outcomes Quality Initiative guideline established that hypertension in hemodialysis patients is diagnosed when pre-dialysis BP is > 140/90 mmHg or when post-dialysis BP is > 130/80 mmHg,^24^ but the conventional peridialytic BP recordings may not be accurate. Pre- and post-dialysis BPs measures are obtained by the staff of the dialysis unit, often without the necessary attention to the correct measurement technique.^1^^,^^2^ Additionally, other factors may dictate an inaccurate pre- and post-dialysis BP reading, such as the white-coat effect, fear of incorrect arteriovenous fistula needling, fluctuations in volume status, and limited time for relaxation (patient is anxious to start dialysis).^25^ Furthermore, the poor diagnostic accuracy of peri-dialytic BP recordings was well stablished by a meta-analysis showing that both pre- and post-dialysis BP readings provide imprecise estimates of the mean interdialytic BP recorded by 44-hour ambulatory BP monitoring.^26^ Thus, an alternative could be the use of the intradialytic BP measurement average, which may provide greater sensibility and specificity in detecting interdialytic hypertension compared to pre- and post-dialysis BP evaluations.^27^

However, BP measurements obtained outside dialysis units are frequently needed to diagnose hypertension in dialysis patients. Home BP monitoring is widely applied and strongly recommended for diagnosis and treatment of hypertension in the general population.^28^ Additionally, home BP was shown to have high short-term reproducibility from one week to the next and it is strongly associated with indices of target organ damage, such as aortic stiffness and left ventricular hypertrophy (LVH).^29^ Currently, many authors suggest that ambulatory BP monitoring (ABPM) may be the gold standard method for diagnosing hypertension in patients receiving dialysis.^2^^), (^12 Observational studies clearly suggest that ABPM predicts all-cause and cardiovascular mortality better than peri-dialytic BP.^13^ ABPM has the advantage of recording BP at night, because many dialysis patients present a non-dipping nocturnal BP pattern that is associated with LVH and cardiovascular mortality.^30^ However, ABPM is inconvenient for many dialysis patients with a high treatment burden, high prevalence of sleep disorders and, eventually, compromised bilateral upper limbs with arteriovenous fistula. Therefore, home BP monitoring appears to be a simple and effective approach to evaluate BP and make therapeutic decisions in dialysis patients.^31^ Table 1 presents information for diagnosis of hypertension in dialysis patients. In contrast to the typical decline in BP during hemodialysis session, 10 to 15% of hemodialysis patients exhibits a paradoxical intradialytic BP elevation^32^ and although this abnormal response has been long recognized, the exact reason for is still not well known. Intradialytic hypertension may be defined as a rise of at least 15 mmHg in mean BP during dialysis or a rise of at least 10 mmHg in systolic BP during or immediately post-dialysis in a certain number of dialysis sessions (the last three or four dialysis sessions).^33^

**Table 1:** Diagnosis of hypertension in dialysis patients

Hypertension in dialysis patients should be based on home BP or ABPM evaluation.
● Home BP in hemodialysis: an average BP ≥ 135/85 mmHg obtained over 6 non-dialysis days, during a two-week period, with the measurements made in a quiet room, with the patient in seated position, back and arms supported, after 5 minutes of rest and with 2 measurements taken 1-2 minutes apart.
● Home BP in peritoneal dialysis: an average BP ≥ 135/85 mmHg over 7 consecutive days with the above described conditions.
● ABPM in hemodialysis patients: an average BP ≥ 130/80 mmHg over 24-hour monitoring during a mid-week non-dialysis day and, if possible, extended to 44 hours.
● ABPM in peritoneal dialysis: an average BP ≥ 130/80 mmHg over 24-hour monitoring.
● For hemodialysis patients: when neither ABPM nor home BP measurements are available, the diagnosis can be made based on office BP measurements taken in a mid-week non-dialysis day, with the standard technique described above.
● For peritoneal dialysis patients: office BP ≥ 140/90 mmHg obtained with the standard technique as described above.

Adopted from Sarafidis et al.^5^

### Mechanisms involved in blood pressure alterations in stage 5 CKD

**Figure 1:**
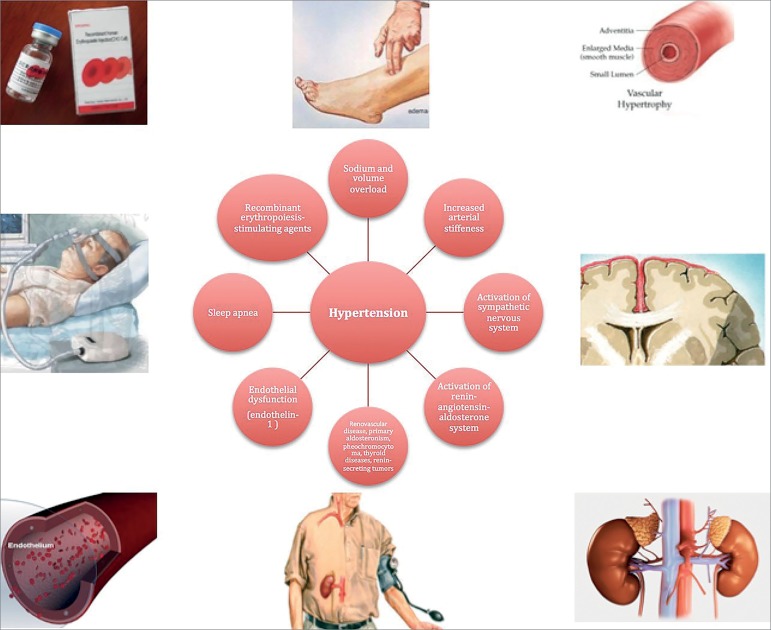
Factors involved in the development of hypertension in dialysis patients.

The pathophysiology of hypertension in dialysis patients is complex and multifactorial.^6^^,^^12^ A selection of risk factors potentially involved in the development of hypertension in dialysis patients is listed in Figure 1. Increase in cardiac output, peripheral vascular resistance, or both result in BP elevation in dialysis patients. First, excessive intravascular volume is a major pathogenic factor of hypertension in dialysis patients and this extracellular volume expansion is most likely to be observed in hypertensive end-stage renal disease (ESRD) patients.^5^ Total body water is increased in hypertensive hemodialysis patients when compared to normotensive^34^ and when excessive body fluids are removed and "dry-weight" is achieved with slow and more frequent dialysis, BP can be ameliorated in approximately 90% of patients.^35^ Indeed, perturbations in vascular auto-regulation may occur in hypervolemic ESRD patients, namely the inappropriate increase in angiotensin II in relationship to volume, increased vascular reactivity to endogenous pressors, and increased cardiac output in the presence of high peripheral vascular resistance.^36^ In many cases, hypertension is related to weight gain during the interval between two dialysis sessions and BP may be ameliorated by correcting the extracellular volume, although the results obtained in different studies are contradictory. In fact, a few studies observed that volume status affects interdialytic BP^37^ while other series failed to confirm this relationship.^38^ Additionally, there is a correlation between loss of weight during hemodialysis and lowering SBP, 39 and volume sensitivity is higher in hypertensive compared to normotensive dialysis patients.^40^

The normalization of the patient's extra-cellular volume is also reported to improve the circadian BP rhythm, which may be abnormal in the presence of volume expansion.^41^ In patients who remain hypertensive despite intensive ultrafiltration, sodium and volume excess may play only a secondary role. Additionally, the lack of correlation between extracellular volume and BP in these patients has been previously described.^42^ Interestingly, Titze et al.^43^ recently described an unknown sodium storage system particularly bound to glycosaminoglycans in skin that does not promote osmotic activity. This novel compartment, at sodium concentrations of 180-190 meq/L, acts as a buffer to exogenous sodium. Inappropriately, this sodium store could be released into the blood, resulting in hypervolemia and oxidative stress or inducing the activation of cellular mechanisms involved in tissue fibrosis. Indeed, in hemodialysis patients, sodium and water in skin and muscle are increased and vascular endothelial growth factor (VEGF) is reduced when compared to age-matched healthy individuals, and this phenomenon may contribute to hypertension.^44^

The role of excessive renin secretion in relation to volume status and sodium has been recognized as an important factor in the pathogenesis of hypertension in dialysis patients. It is well-known that activation of the renin-angiotensin-aldosterone system occurs even in ESRD patients in dialysis treatment,^45^ eventually resulting in dialysis refractory renin-dependent hypertension. Additionally, secondary hyperaldosteronism contributes to hypertension and it has recently become clear that apart from hypertension, aldosterone may have numerous blood-pressure-independent actions that under conditions of high salt concentration, is injurious to the kidney, heart, and vasculature.^46^

Increase arterial stiffness occurs frequently in dialysis patients, mainly related to calcium and phosphate disturbance metabolism resulting in vascular calcification.^47^ Premature vascular aging and arterial stiffening are observed with progression of CKD and in ESRD. This accelerated aging is associated with outward remodeling of large vessels, characterized by increased arterial radius that is not totally compensated for by artery wall hypertrophy. Arterial stiffening in CKD and ESRD patients is of multifactorial origin with extensive arterial calcifications representing a major covariate.^48^ In dialysis patients, arterial stiffness assessed by aortic pulse wave velocity (PWV) is closely related to high interdialytic BP, and increasing PWV blunts the circadian amplitude of systolic BP and pulse pressure.^49^

Increased activity of the sympathetic nervous system may contribute to hypertension in ESRD patients.^50^ Sympathetic nerve discharge was 2.5 times higher in dialysis patients than in normal subjects and this discharge was not correlated with either plasma noradrenaline concentration or plasma renin activity.^51^ Fluid overload of greater than 6% of body weight results in activation of the sympathetic nervous system,^52^ and angiotensin-converting enzyme (ACE) inhibition could result in reduction of this sympathetic hyperactivity.^53^ Endothelium-dependent vasodilatation is impaired in uremia, and nitric oxide (NO) deficiency occurs in ESRD patients, contributing to hypertension in hemodialysis and peritoneal dialysis patients.^54^ The production of NO by the vascular endothelium is inhibited by asymmetric dimethylarginine (ADMA), which accumulates in CKD patients, particularly in those with atherosclerotic complications.^55^ However, no significant correlation was observed between ADMA concentrations and BP in dialysis patients.^34^ Additionally, deficiency of renalase, an enzyme produced by the kidney that metabolizes catecholamines and catecholamines-like substances, may contribute to increased sympathetic nervous system activity in CKD.^56^

Endothelial dysfunction may contribute to hypertension in dialysis patients through several mechanisms. Patients with CKD show reduced NO availability measured as NO-dependent vasodilatation and this phenomenon may be related to reduced NO production.^57^ Indeed, high circulating levels of asymmetric dimethylarginine (ADMA), an endogenous NO synthase inhibitor, are observed in CKD patients, 58 and in ESRD hemodialysis patients, ADMA is associated with cardiovascular disease and mortality. 59 Additionally, endothelin-1 may have an important role in the development of intradialytic hypertension, 60 which occurs regularly in 10-15% of hemodialysis patients.^61^

In 20 to 30% of CKD patients, regular administration of human recombinant erythropoietin (rHuEPO) is accompanied by "de novo" hypertension or aggravation of preexisting hypertension, and the increase in BP occurs within a few weeks to months after initiation of rHuEPO.^62^ Grekas et al.^40^ observed hypertension in 62% of rHuEPO-treated hemodialysis patients but only in 38% of those not receiving rHuEPO. An increase in red cell mass during or after correction of anemia leads to increase whole-blood viscosity and cardiac afterload^63^ and may contribute to hypertension in those patients, but increase in BP may occur even before hematocrit increases.^64^ Other factors related to rHuEPO-induced hypertension in CKD patients include endothelin release, vascular endothelial dysfunction, preexisting hypertension, elevation of cytosolic free calcium in vascular smooth muscle cells, inhibition of NO synthesis, and rapid correction of anemia.^65^ Additionally, higher rHuEPO doses, higher target hemoglobin levels,^66^ and possibly dialysis modality^67^ have been associated with a higher BP response.

Sleep apnea is highly prevalent and may be related to volume overload^68^ in dialysis patients. Nocturnal hypoxemia in sleep apnea has been associated with higher nocturnal SBP and left ventricular relative wall thickness,^30^ and resistant hypertension,^12^^,^^69^ while the obstructive apnea-hypopnea index is significantly reduced after hemodialysis with reduction of fluid overload.^70^

Secondary hyperparathyroidism may also result in hypertension in ESRD population by mechanisms including entry of calcium into vessel wall smooth muscle cells. However, parathyroidectomy failed to correct hypertension in patients on chronic hemodialysis.^71^ In contrast, activated vitamin D therapy for secondary hyperparathyroidism resulted in significant decreases in mean BP.^72^

In dialysis patients, plasma α-human atrial natriuretic peptide (α-ANP) levels are elevated, reflecting extracellular volume expansion. The α-ANP values decrease post-dialysis but remain elevated in patients with altered left atrial hemodynamics (65). Similar to α-ANP, the concentration of brain natriuretic peptide (BNP) is higher in hemodialysis patients than in healthy volunteers, and BNP is lowered less efficiently by dialysis procedure.^73^ Franz et al.^74^ observed that, in hemodialysis patients with moderate or severe hypertension, the levels of pro-ANP fragments and α-ANP were higher than in patients with mild hypertension. Indeed, cardiac natriuretic peptides are related to left ventricular mass and predict cardiovascular mortality in dialysis patients.^6^

Although it has been established that interdialytic salt restriction or intradialytic removal of salt and fluids is effective in reducing BP, success over time is very rare.^75^ Other studies have been performed in dialysis population to investigate the impact of salt restriction on blood pressure levels. Ozkahya et al.,^76^ by emphasizing sodium restriction, stopping all antihypertensive drugs, and intensifying ultrafiltration, observed not only significant reduction on BP levels, but also in left ventricle wall thickness. The same group observed, in another study, that sodium chloride restriction to < 6 g/day determined normalization of BP levels after 36 months.^77^

Because the sodium concentrate of the dialysate is usually higher than that of the patient's serum, it can influence post dialysis thirst, interdialytic weight gain, and BP. In addition, salt balance is positive with the habitual high dietary sodium intake and use of saline solutions to maintain plasma volume during UF and to treat hypotension episodes during dialysis treatment. Low sodium level in dialysate resulted in lower intra- and inter-dialytic plasma sodium when compared with high dialysate sodium,^78^ and a programmed variable sodium dialysis from 155 meq/L to 135mEq/L resulted in a reduction of antihypertensive drugs use, without alterations in predialytic BP when compared to a dialysate sodium concentration of 140 meq/L.^79^

### Management of hypertension in stage 5 CKD

Current data from several observational studies^12^^,^^80^ and a prospective cohort study^81^ suggest a "U-shaped" association between pre-HD BP and mortality. This means that blood pressure below certain levels may be more harmful than high levels, especially when patients present with severe cardiomyopathy, that often modifies the relationship between BP and mortality, determining a very low survival in ESRD patients with SBP < 115 mmHg (82). On the other hand, post-dialysis SBP > 180 and DBP > 90 mmHg were associated with increase in cardiovascular mortality and should be treated aggressively.^83^ This reverse epidemiology of BP and cardiovascular mortality makes it difficult to establish a real and reliable target for BP levels in dialysis patients. Nevertheless, international guidelines for cardiovascular disease recommend BP level less than 140/90 mmHg at the beginning of the week. However, this recommendation should not be applied uniformly in the dialysis setting,^12^^,^^84^ because the aggressive approach to control BP can increase the risk of symptomatic intra-dialytic hypotension and its consequences.

### Non-pharmacological therapy

Most patients in stage 5 CKD develop a positive sodium balance and an increase in extracellular volume (ECV), with salt and water overload playing a central role in the development of hypertension. High salt intake has been shown to be associated with high pre-dialysis SBP and cardiovascular disease.^85^ Normalizing sodium and fluids balance is key to control BP and to reduce cardiovascular events, as stated by the most recent guideline;^6^ dietary salt restriction should be below 5-6 g/day and interdialytic weight gain should not exceed 0.8 kg/day. Indeed, in peritoneal dialysis patients, salt and water excess are the most important determinants of elevated BP^12^^,^^86^ and many authors recommend salt restriction (< 5g/day) for all peritoneal dialysis patients unless there is evidence of volume contraction.^87^ Such dietary targets are particularly important in the presence of loss of residual renal function and when the patient have a high membrane transport that negatively interfere in the ultrafiltration.

Another way to regulate the fluid volume of dialysis patients, particularly in hemodialysis, is to set an appropriate dry weight (DW). In clinical practice, the DW is usually established by a progressive decline in post dialysis body weight over a 4-8-week period after initiation of maintenance hemodialysis (88). This post-dialysis DW may be defined as the post dialysis body weight at which ECV in within the normal range or the target BP value without the need for antihypertensive medications.^6^ These definitions, obviously, cannot be applied to those patients who are hypotensive because of cardiomyopathy. In contrast, establishing a DW for PD patients is very complicated and the motive of frequent debates. There are some attempts to monitor volume status of PD patients with multifrequency bioimpedance and the results are acceptable.^12^^,^^89^

The clinical history and physical examination may help in detecting more obvious ECV increases, but in general, assessment of DW using clinical parameters presents low sensitivity.^90^ Attempts have been made to determine DW by bioimpedance device (BIA)^91^ by monitoring regional resistance and resistivity in the calf, showing that the prescribed target weight can be decrease over time, improving BP control. Other BIA devices that assess whole body composition provide readouts of BP and ECV status that may be helpful in the follow-up of fluid balance and information about increased risk of mortality when overhydration is present.^92^ Randomized control studies have demonstrated that optimization of DW by bioimpedance methods are safe and capable of improving BP control in dialysis patients.^12^^,^^93^

Other methods for assessing ECV include measurement of vena cava diameter, which requires time for post dialysis equilibration and is operator-dependent.^94^ Lung ultrasound can detect asymptomatic pulmonary congestion in hemodialysis patients, and the resulting BL-US (B-lines ultrasound) score is a strong and independent predictor of death and cardiac events in this population.

Increasing the dialysate sodium concentration above the pre-dialysis values may help reduce episodes of intradialytic hypotension but may lead to increased weight gain by enhanced thirst.^6^ It may be best to adjust sodium dialysate concentration to match the patient's pre-dialysis plasma sodium and not use higher dialysate sodium. Use of hypertonic dextrose rather than saline in the management of intradialytic hypotension and cramps also increases the potential for a neutral sodium balance. Dietary salt restriction is useful for DW optimization and blood pressure control in dialysis patients. Several studies have consistently reported a decrease in interdialytic weight gain, associated reduction in BP levels, and more significant reduction in left ventricular mass.^12^^,^^96^

### More frequent dialysis sessions

Conventional hemodialysis is frequently associated with high ultrafiltration (UF) rates, which enhance the risk of muscle cramps and hypotensive episodes. The symptoms are treated by saline intravenous infusion, which favors an expanded ECV, hypertension, and risk of developing LVH. The prescription of longer or more frequent dialysis sessions allows the decrease in UF rates and reduces the risk of intradialytic complications,^97^ improving LVH^98^ and cardiac function.^99^ More frequent hemodialysis sessions than the conventional three times weekly regimen reduces BP more consistently and requires fewer anti-hypertensive medications to achieve the same BP control.^12^^,^^100^ The European Best Practice guidelines recommend that the length of the hemodialysis session should not be determined only by an optimal KT/V result, but by stablishing at least three dialysis sessions of 4 hours each to ensure optimal volume status.^101^

Additionally, in the FREEDOM trial,^102^ a prospective cohort study of short daily HD, the mean number of prescribed antihypertensive agents decreased from 1.7 to 1.0 in 1 year, whereas the percentage of patients not prescribed antihypertensive agents increased from 21 to 47%. Kotanko et al.^103^ analyzed the effects of more frequent hemodialysis sessions on BP control in a randomized controlled trial, including patients on daily diurnal and nocturnal HD treatment versus conventional three weekly HD sessions and observed after twelve months a sustained and significant reduction in both diastolic and systolic BP, as well in the number of prescribe antihypertensive medications. Nocturnal HD appears to markedly reduce total peripheral resistance and plasma norepinephrine and restore endothelium-dependent vasodilation. In conclusion, the above information indicate that intensive HD, in general, reduces BP and the need for antihypertensive medications.

### Pharmacological therapy

When prescribing antihypertensive drugs to stage 5 CKD patients on dialysis one must be aware that pharmacokinetics may be altered by the impaired kidney excretion and the drug dialyzability. In addition, reduced compliance, side effects, and financial costs can have an impact in treatment effectiveness. Other problems related to this special population are the occurrence of intradialytic hypotension and vascular access thrombosis.^104^ Moreover, some antihypertensive effects drugs are also cardioprotective, decreasing the risk of death by cardiovascular disease. Examples of drugs in this category are the renin-angiotensin-aldosterone system (RAAS) inhibitors, β-adrenergic blockers, calcium channel blockers (CCBs), and aldosterone inhibitors (for patients not on dialysis). Angiotensin II has been implicated in endothelial dysfunction, smooth muscle proliferation, atherosclerotic plaque rupture, and LVH, the latter occurring even when BP is controlled.^6^ In the general population, the use of RAAS decreases cardiovascular events^105^ in patients with left ventricular dysfunction and in stable coronary artery disease. Similarly, in the non-ESRD population, the clinical use of β-blockers confer cardiovascular protection^106^ and CCBs decrease intracellular calcium levels produced by secondary hyperparathyroidism and alter lipid profile, which may reduce cardiovascular risk^107^.

Studies of antihypertensive drugs in CKD dialysis patients have shown limited results and two meta-analysis of randomized trials conclude that the real merit of these drugs (RAAS, CCBs, β-adrenergic blockers) are not well established.^108^^,^^109^ The two meta-analyses confirmed that treatment with antihypertensive agents was associated with reduction on cardiovascular events, but when normotensive patients were included in the analysis, the beneficial effects of the drugs were markedly diminished, becoming non-significant. Most importantly, none of the trials included in these meta-analyses specifically targeted BP levels. According to these data, BP control by anti-hypertensive drugs leads to better cardiovascular outcomes, however, an optimal regimen to control BP and reduce mortality has not yet been stablished.

As for hemodialysis, there is a lack of studies to define the ideal target for blood pressure to reduce cardiovascular events. However, one recent study deserves some comments. A randomized controlled trial described a significant benefit in the cardiac function of peritoneal dialysis patients with the use spironolactone in addition to a RAAS inhibitor without any additional risk of hyperkalemia.^110^ Finally, there are not enough studies comparing the benefits of one class of antihypertensive over another for PD patients. Nevertheless, the positive impact of the RAAS inhibitors on the residual renal function and in the preservation of the peritoneal membrane found in some studies gave some popularity to these classes of antihypertensive drugs.^111^^,^^112^

Recommendations on antihypertensive drugs in CKD dialysis patients are based on their effects in BP reduction, side effects, and protective cardiovascular effects. The use of RAAS inhibitors, β-adrenergic blockers, and CCBs is desirable in dialysis patients because of their effects on plasma renin activity, in reducing sympathetic activity, and in decreasing intracellular calcium levels, respectively.^6^ Beyond any individual preference, there is no strong evidence to recommend one specific class of antihypertensive drug over another in CKD dialysis population and only few clinical trials have demonstrated some beneficial cardiovascular effects of RAAS inhibition and β-adrenergic blockers in those patients.^113^^,^^114^ RAAS inhibitors should be used in CKD-VD patients because these agents are particularly beneficial for cardiac disease frequently observed in dialysis patients and are effective in reducing left ventricular mass and mortality.^115^^,^^116^ Specifically related to this topic of interest, there is an ongoing phase 3 trial evaluating Spironolactone 25 mg (Aldosterone bloCkade for Health Improvement EValuation in End-stage Renal Disease (ACHIEVE) - https://clinicaltrials.gov/ct2/show/NCT03020303) and its purpose is to determine if spironolactone reduces death or hospitalization for heart failure and if the drug is well tolerated in patients that require dialysis.

Hypertension and heart failure (HF) are conditions frequently seen in the CKD population and sympathetic overactivity plays an important role in this scenario, making β-blockers suitable for treating both conditions (117). A meta-analysis concluded that treatment with β-blockers improved all-cause mortality in patients with CKD and heart failure (118). Additionally, some prospective studies demonstrated that the use of β-blockers are associated with reduce risk of mortality in hemodialysis patients.^114^^,^^119^ More recent, another therapeutic agent, sacubitril-valsartan, was approved for use in patients with HF and this dual-acting agent enhances the functions of natriuretic peptides and inhibits the renin-angiotensin system,^120^ with potential benefit for CKD patients.

Finally, the removal of an antihypertensive drug during dialysis sessions (for example β-blockers) may predispose patients to uncontrolled BP^121^ and the pharmacokinetic of ACE inhibitors are quite different among each other, determining the post-dialysis supplementation of drugs in some cases. Some drugs with long-acting antihypertensive effects (Atenolol and Lisinopril) can be administered thrice weekly, thus enhancing pharmaco-adherence.^12^^,^^122^

## Conclusions

Hypertension is frequently diagnosed in the dialysis population, difficult to manage, and associated with an increased risk of cardiovascular disease. The complex pathophysiology of this condition explains the great difficulty of its treatment. At present, the superiority of home self-measured blood pressure over pre-hemodialysis is convincing and other investigation tools, like ambulatory blood pressure monitoring, are becoming more applied in CKD populations. In general, all antihypertensive drugs can be used in dialysis population, with the adequate dose adjustment determined by clearance during dialysis sessions. The use of combined non-pharmacologic, particularly dietary sodium restriction, dialysate sodium adjustment and use of antihypertensive drugs (preferentially cardioprotective ones) may be the best practice to optimize blood pressure control. Randomized clinical trials with anti-hypertensive drugs aiming to reduce mortality are still needed, as well as a definitive guideline of BP control in dialysis population. In addition, non-pharmacological interventions with different dialysis modalities or schemes and sodium restriction should be adequately tested in this high-risk population.
